# Information encoding and encryption in acoustic analogues of qubits

**DOI:** 10.1038/s41598-024-65800-z

**Published:** 2024-06-28

**Authors:** Akinsanmi S. Ige, David Cavalluzzi, Ivan B. Djordjevic, Keith Runge, Pierre A. Deymier

**Affiliations:** 1 Department of Materials Science and Engineering, The University of Arizona, Tucson, AZ 85721 USA; 2 Department of Electrical and Computer Engineering, The University of Arizona, Tucson, AZ 85721 USA; 3 New Frontiers of Sound Science and Technology Center, The University of Arizona, Tucson, AZ 85721 USA

**Keywords:** Cryptography, Data encryption, Acoustic metamaterials, Quantum analogue, Engineering, Information theory and computation

## Abstract

Cryptography is crucial in protecting sensitive information and ensuring secure transactions in a time when data security and privacy are major concerns. Traditional cryptography techniques, which depend on mathematical algorithms and secret keys, have historically protected against data breaches and illegal access. With the advent of quantum computers, traditional cryptography techniques are at risk. In this work, we present a cryptography idea using logical phi-bits, which are classical analogues of quantum bits (qubits) and are supported by driven acoustic metamaterials. The state of phi-bits displays superpositions similar to quantum bits, with complex amplitudes and phases. We present a representation of the state vector of single and multi-phi-bit systems. The state vector of multiple phi-bits system lies in a complex exponentially scaling Hilbert space and is used to encode information or messages. By changing the driving conditions of the metamaterial, the information can be encrypted with exceptional security and efficiency. We illustrate experimentally the practicality and effectiveness of encoding and encryption of a message using a 5 phi-bits system and emphasize the scalability of this approach to an *N* phi-bits system with the same processing time.

## Introduction

Protecting the confidentiality and integrity of data, as well as securing transactions and communications is crucial in government and non-governmental organizations. Evidently, this has been accomplished by the utilization of cryptography, which involves the encryption and decryption of data by converting it from its original form to an unreadable format utilizing a secret key and vice versa^1^. It addresses the issues of data leakage and ensures that only authorized individuals can access the information. It gives information immunity against attacks and unauthorized access and manipulation. Classical cryptography uses mathematical operations to encode messages and may employ two types of key encryption algorithms to encrypt and decrypt the information^1–3^. The advent of quantum computers poses a significant threat to the field of cryptography due to their remarkable computing speeds and ability to decipher many encryption algorithms currently in use^4,5^. However, the introduction of post-quantum cryptography (PQC) protocols, such as those based on lattice encryption, has demonstrated effectiveness in fortifying classical encryption against quantum attacks^3^. A quantum computer leverages the ability of its processing units (qubits) to exist in the superposition of states to execute parallel computation. Quantum computers can efficiently solve mathematical problems that are computationally impractical for classical computers. This includes solving the factorization problem, which is one of the bases of classical encryption. Furthermore, it has been demonstrated that other current classical encryption systems may be bypassed using straightforward quantum techniques^6,7^. The National Institute of Standards and Technology (NIST) predicts that quantum computers will render present public key encryption techniques obsolete by 2028. It is believed that further advancement in the quantum computer will totally eradicate classical encryption^4,7^. In that context, researchers are focusing on post-quantum cryptography (PQC) to prepare for the anticipated future risk posed by quantum computers. PQC’s objective is to create novel algorithms capable of withstanding quantum attacks, which integrate quantum algorithms into the existing classical encryption framework^3,5,7,8^.

In a conventional computer, a classical bit encodes information as either a 0 or a 1, whereas, the quantum equivalent of a classical bit, qubit, can exist simultaneously in a coherent superposition of the quantum basis states $\left|0\right.\rangle$ and $\left|1\right.\rangle$^9^. Even though some quantum algorithms (quantum circuits) can be efficiently simulated using classical computers^10^, the anticipated advantage of quantum computing lies in its massively parallel processing capabilities associated with quantum superposition and entanglement. The quantum wavefunction represents a probability amplitude, implying that a system composed of multi-qubit systems is highly vulnerable to noise and wavefunction collapse triggered by any disturbance in the quantum system, which scales exponentially with an increase in the qubit system^11,12^. This presents a significant challenge in the experimental realization of multi-qubit systems. To address the scalability difficulties in quantum computing, we introduce a method for encrypting and decrypting data using multiple logical phi-bit systems. A phi-bit is a classical analogue of a quantum bit supported by a driven acoustic metamaterial constituted of parallel acoustic waveguides^9^. A phi-bit is specifically associated with a two-state degree of freedom of an acoustic wave, which can be in a coherent superposition of states with complex amplitude coefficients. A phi-bit like its counterpart qubit exhibits superposition and entanglement of states which is the foundation of quantum computing^11^.

The concept of phi-bit was recently broadened from the physical to the logical domain^11,13^. A logical phi-bit is conceptualized as a two-level system characterized by complex amplitudes analogous to a qubit. Notably, phi-bits co-exist in the same physical space which indicates independent of the phi-bit scalability on distance. Logical phi-bits exploit the strong coupling and nonlinearity of acoustic waves to realize non-separable superpositions of states spanning exponentially complex spaces (i.e., Hilbert space), a prerequisite to developing algorithms that exploit the computational parallelism arising from non-separability and hence can be employed for programming. The importance of the representation of multiple phi-bit vector states as the result of changes in the Hilbert space basis has been emphasized in^9^.

This study presents a novel approach to data encryption utilizing logical phi-bits, which are generated through the experimental setup of three aluminum rods arranged in a linear array with transducers to drive and detect acoustic fields. These phi-bits are nonlinear vibrational modes represented by frequency and relative phases between waveguides, similar to qubits in quantum computing. The paper illustrates the simultaneous manipulation of phi-bits by controlling the conditions, enabling efficient encryption and decryption of information. The encryption process involves encoding a message into the state vector of phi-bits and applying unitary transformations, such as changes in driving frequencies, to effectively encrypt and decrypt the message. The scalability of the system is highlighted, with encryption processing time remaining constant regardless of the number of phi-bits used. While we recognize that an extensive security analysis, including the transmission of the message, is crucial in the field of cryptography. However, in this study, our focus is demonstrating the feasibility of an encryption scheme using a phi-bit state vector and presents logical phi-bits as a promising solution for encryption in the digital age. The security analysis is beyond the scope of this paper and will be addressed in future work. The paper concludes by emphasizing the importance of adapting cryptographic methods to advancements in technology, with logical phi-bits offering a reliable approach to strengthening data security.

## Logical phi-bit and representation and proposed encryption scheme

A logical phi-bit was generated by replicating the experimental arrangement described in^10^, the setup consisted of three aluminum rods, approximately 60 cm long, arranged in a linear array with a lateral gap of 2 mm filled with epoxy. Transducers were used to drive and detect the acoustic field at the ends of the rods. The waveguides are labeled 1, 2, and 3, where waveguide 2 is sandwiched between waveguides 1 and 3. Two driving transducers located on waveguide 1 and waveguide 2 were excited with sinusoidal signals at the primary frequencies ${f}_{1}$ and ${f}_{2}$, respectively. The third waveguide was not driven. The driving transducers were connected to different function generators and amplifiers and were driven with the same peak-to-peak voltage of 80 V. Three detecting transducers were connected to an oscilloscope to measure the voltage (displacement field) generated at the other ends of the waveguides. The detected temporal signals are fast Fourier transformed (FFT) to produce a power spectrum. In the spectral domain, we observed strong peaks associated with the primary frequencies as well as weaker peaks associated with nonlinear vibrational modes supported by the inherently nonlinear system. A logical phi-bit,’*i*’, is defined as a nonlinear mode in which frequency can be written as a linear combination of the primary frequencies:

1 $${F}^{(i)}=p{f}_{1}+q{f}_{2}$$

where *p* and *q* are integer coefficients.

A single logical phi-bit ‘*i*’ is characterized by its frequency but also the corresponding relative phases between the acoustic waveguides. The relative phases between waveguides 1 and 2 and between waveguides 1 and 3 at the phi-bit frequency, ${F}^{(i)}$, are written as the phase difference ${\varphi }_{12}^{(i)}={\varphi }_{2}^{(i)}-{\varphi }_{1}^{(i)}$ and ${\varphi }_{13}^{(i)}={\varphi }_{3}^{(i)}-{\varphi }_{1}^{\left(i\right)}$, respectively. Here, the phase of the nonlinear mode at the end of waveguide 1 is used as a reference.

The two-phase differences serve as degrees of freedom for representing a logical phi-bit as a two-level system that may be characterized as a 2 × 1 vector with complex components. Within this representation, a logical phi-bit is analogous to a qubit. Notably, logical phi-bits co-exist in the same physical space, that is, the space of the physical system. Since logical phi-bits are nonlinear vibrational modes generated from the same driving frequencies, their associated phase differences are correlated to each other. Subsequently, the two-level system representations of multiple phi-bits are also strongly correlated and can be simultaneously manipulated by tuning the driving conditions of the system. Effectively, the state of *N* logical phi-bits may be represented as a 2^*N*^ × 1 complex vector spanning an exponentially scaling space (i.e., a Hilbert space). The representation of the *N* phi-bit state depends on the choice of the basis of the Hilbert space. The strong nonlinear coupling between the phi-bits enables manipulation of the component of this large complex vector in a parallel manner by changing the driving conditions. Furthermore, the components of the large multi-phi-bit state vector can be used to encode information. Parallel manipulation of these components can be used to encrypt the encoded information. The inverse manipulation can also be used to decrypt the encrypted information in an efficient manner.

We illustrate this encryption method in the case of a *N* = 5 phi-bit system. For this, the driving frequencies are taken as ${f}_{1}=62\,\text{kHz}$ and ${f}_{2}=66\,\text{kHz}$. We tune ${f}_{1}$ by increments of $\Delta \nu \left(n\right)=\left(n-1\right)*50\,\text{Hz}$ with $n\in [\text{1,81}]$, thus spanning a range of frequency of 0 to 4kHz.

**Figure 1:**
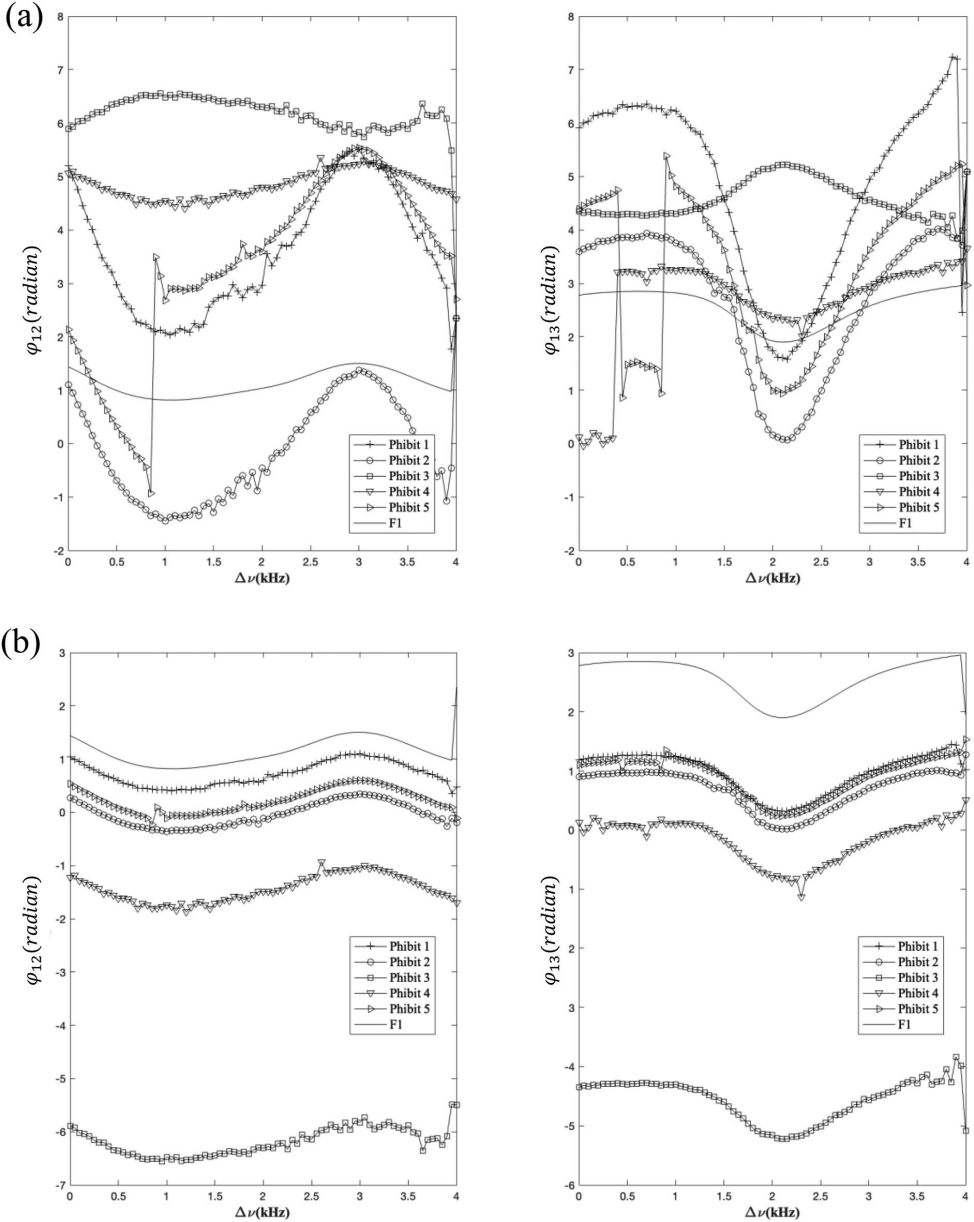
(**a**) Experimentally measured ${\varphi }_{12}^{\left(i\right)}(\Delta \nu )$ and ${\varphi }_{13}^{\left(i\right)}(\Delta \nu )$ for the five phi-bits *i* = 1…,5. (**b**) Rescaled and corrected ${\varphi }_{12}^{\left(i\right)}(\Delta \nu )$ and ${\varphi }_{13}^{\left(i\right)}(\Delta \nu )$. See text for details.

We are considering phi-bits 1 through 5 with corresponding values *p* and *q* of (5, − 4), (4, − 3), (− 1, 2), (1, 1), and (4, − 1), respectively. The phi-bits are selected at random which indicates randomness of the *p* and *q* values. In Fig. 1a, we report experimentally measured ${\varphi }_{12}^{\left(i\right)}(\Delta \nu )$ and ${\varphi }_{13}^{\left(i\right)}(\Delta \nu )$ for the five selected phi-bits. We note that the phase differences exhibit two different behaviors. The first behavior is associated with a common monotonous variation of the phase differences. The second behavior which is only occurring in the case of phi-bits 4 and 5 takes the form of sharp 180° ($\pi$) jumps. The possible physical origin of these two behaviors has been discussed at length in references^14^. Here we focus on the first behavior by correcting the phase differences by the $\pi$ jumps to obtain continuous variations for all phi-bits. Moreover, in the case of the first behavior we have shown in^15^ that the phase differences of the phi-bits relate to the phase differences at the primary frequencies in a linear manner. Indeed, we can write.

2a $${\varphi }_{12}^{\left(i\right)}\left({f}_{1}+\Delta \nu ,{f}_{2}\right)=p{\varphi }_{12}\left({f}_{1}+\Delta \nu \right)+q{\varphi }_{12}\left({f}_{2}\right)$$

2b $${\varphi }_{13}^{\left(i\right)}\left({f}_{1}+\Delta \nu ,{f}_{2}\right)=p{\varphi }_{13}\left({f}_{1}+\Delta \nu \right)+q{\varphi }_{13}\left({f}_{2}\right)$$

where *p* and *q* are the same coefficients as in Eq. (1).

In Fig. 1b, we rescale the corrected phase differences of the phi-bits by the factor *p* only, since the ${\varphi }_{12}\left({f}_{2}\right)$ and ${\varphi }_{13}\left({f}_{2}\right)$ remain constant. The rescaled and corrected phase differences of the five phi-bits behave simultaneously in the same manner. The vertical offset is simply due to the constant ${q\varphi }_{12}\left({f}_{2}\right)$ and ${q\varphi }_{13}\left({f}_{2}\right)$.

Each phi-bit, “*i*”, is represented in a two-dimensional Hilbert space, taking the form reminiscent of a Bloch sphere representation:

3 $$V^{\left( i \right)} = \left( {\begin{array}{*{20}c} {\sin \left( {\beta^{\left( i \right)} } \right)} \\ {e^{{i\gamma^{\left( i \right)} }} \cos \left( {\beta^{\left( i \right)} } \right)} \\ \end{array} } \right).$$

In that representation, for the sake of simplicity, we assume $\gamma^{\left( i \right)} = 0$*,* and

4 $$\beta^{\left( i \right)} \left( {\Delta \nu \left( n \right)} \right) = K\left( {\varphi_{12}^{\left( i \right)} \left( {\Delta \nu \left( n \right)} \right) - \varphi_{12}^{\left( i \right)} \left( {\Delta \nu \left( {n = 1} \right)} \right)} \right) + \frac{3\pi }{4}$$

To address the offset in the rescaled and corrected phases of Fig. 1b we use ${\varphi }_{12}^{(i)}\left(\Delta \nu \left(n=1\right)\right)$ as origins as well as the reference for encoding information. In Eq. (4), *K* is a scaling factor to amplify the range spanned by ${\varphi }_{12}^{(i)}$. For the sake of simplicity, we have made ${\beta }^{\left(i\right)}$ a function of ${\varphi }_{12}^{(i)}$ only, other forms could be defined as function of ${\varphi }_{12}^{(i)}$ and ${\varphi }_{13}^{(i)}$ offering an extra degree of freedom in the manipulation of the phi-bit state vector. At the driving frequency ${f}_{1}=62\,\text{kHz} \,\,\text{and}\,\, n=1$, ${\beta }^{\left(i\right)}=\frac{3\pi }{4}$ and

5 $$V^{\left( i \right)} = \frac{1}{\sqrt 2 }\left( {\begin{array}{*{20}c} 1 \\ { - 1} \\ \end{array} } \right).$$

Let us consider a system made of five phi-bits, we can define the state of the system as the tensor product of the phi-bits:

6 $$V = V^{\left( 1 \right)} \otimes V^{\left( 2 \right)} \otimes V^{\left( 3 \right)} \otimes V^{\left( 4 \right)} \otimes V^{\left( 5 \right)} .$$

This tensor product evaluated at the reference driving frequency takes the form:

7 $$V^{T} = \frac{1}{{2^{5/2} }}\left[ {1\,\, - 1\,\,- 1\,\,1\,\,- 1\,\,1\,\,1\,\,- 1\,\,- 1\,\,1\,\,1\,\,- 1\,\,1\,\,- 1\,\,- 1\,\,1\,\,- 1\,\,1\,\,1\,\,- 1\,\,1\,\,- 1\,\,- 1\,\,1\,\,1\,\,- 1\,\,- 1\,\,1\,\,- 1\,\,1\,\,1\,\,- 1} \right].$$

We now consider a new representation of the five phi-bit state vector,

8 $$\widehat{V}= \frac{\left(1+ {V}^{{\prime}T}\right)}{2}= \left[1\,\,0\,\,0\,\,1\,\,0\,\,1\,\,1\,\,0\,\,0\,\,1\,\,1\,\,0\,\,1\,\,0\,\,0\,\,1\,\,0\,\,1\,\,1\,\,0\,\,1\,\,0\,\,0\,\,1\,\,1\,\,0\,\,0\,\,1\,\,0\,\,1\,\,1 0\right]$$

where $${V}^{{\prime}T}={{2}^{5/2}V}^{T}$$.

This new representation of the five phi-bit state corresponds to a change of basis of the Hilbert space. While the vector *V* is separable, the vector $\widehat{V}$ is not separable into a tensor product of single phi-bit states. In quantum information science non-separability of multiple qubit states is necessary for establishing quantum correlations between the qubits. In the case of phi-bit based information science, the components of a multi-phi-bit state vector such as Eq. (7) are correlated via the nonlinearity of the physical system. The string of correlated ones and zeros in $\widehat{V}$ can therefore be employed to encode a message as well as encrypt that message by applying a unitary transformation on $\widehat{V}$. Here we illustrate this encryption process in the case of a short message encoded using 5 phi-bits. In general, the state vector $\widehat{V}$ of a *N* phi-bit system is a 2^*N*^x1 vector with 1 and 0 elements providing sufficient bits for encoding very long messages when *N* is large. For instance, when *N* = 50 the number of bits available for encoding a message is on the order of 10^15^*.* The encryption of a message encoded on this large number of bits would only require a change in the driving condition of the array of waveguides to achieve the encrypting unitary transformation. Such an operation takes approximately a millisecond on the physical system used here, since the speed of sound in the constitutive materials is on the order of a few thousand meters per second.

## Examples of message encoding and encryption

**Figure 2:**
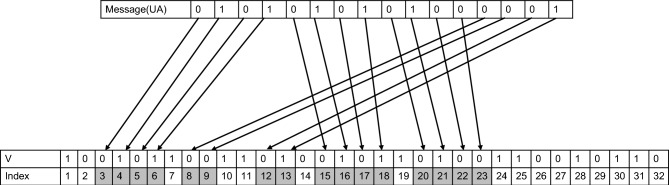
Indexing of the binary message ‘UA’ on a 5 phi-bits state vector.

Let us consider encoding a message such as ‘UA’ using our 5 phi-bits system. The message in its American Standard Code for Information Interchange (ASCII) format is 85 65, which is equivalent to UA = 01010101 01000001 in an 8-bit binary system. The length of the message and the number of ones and zeros in its binary representation determine the number of phi-bits needed for encoding. In this case, 5 phi-bits are sufficient to support the message successfully since the state vector $\widehat{V}$ is 32 components long. Figure 2 illustrates the encoding of the binary form of the message in the vector $\widehat{V}$ by indexing some of its components. The indexing process involves encoding each digit of the message vector into the corresponding index in the state vector, as this encoding proceeds sequentially, any index of the state vector occupied by the message digit will become unavailable for the next digit of the message. It's important to note that this indexing is not necessarily unique and may not be consecutively ordered. The message ‘UA’ is now taking the index form: UA_index_ = [3 4 5 6 15 16 17 18 20 21 22 23 8 9 12 13].

All other components of the phi-bit state vectors are just padding the message.

To encrypt the message, we change the driving condition of the first waveguide to $\left({f}_{1}^{*}={f}_{1}+\Delta \nu (n=20)\right)=62{,}950\,\text{Hz}$ and we use *K* = 2.7 in the representation of Eq. (4). This change in driving condition is equivalent to a rotation of the vector $\widehat{V}$ in the Hilbert space, that is, a unitary transformation. Using the experimentally measured rescaled and corrected ${\varphi }_{12}^{(i)}\left(\Delta \nu =0\right)$ to calculate the numerical value of the encrypted indexed components, we obtain the encrypted message in the form,

The encrypted indexed components are now decimal numbers as shown in Table 1. In quantum information science, another additional advantage of quantum computers over classical computers is the No-Cloning Theorem^16^, which states that creating an exact copy of an unknown quantum system is impossible. This fundamental principle underscores the unique nature of quantum information and highlights the limitations imposed on information processing in quantum systems. This concept is pertinent to the phi-bit system, as it aligns with the inherent constraints and characteristics of quantum information processing, reinforcing the security and integrity of the encoding process. The recipient and the sender of the message need to have the same physical system and must agree on the phi-bit representation, and the number of phi-bits. The encrypted message is sent to the recipient in the following form: the driving frequency ${f}_{1}^{*}$ at which the message is encrypted and UA_index_. This message can be sent over any type of communication channel. The recipient needs to know the key to decrypt the message, this key is effectively the change in driving frequency that is needed to read the original message. Here this key is $-\Delta \nu (n=20)$. However, because of the possibility of small differences in the sender and recipient systems, the decrypted message may not give exact values of zeros and ones for the indexed components. In that case, the decrypted decimal values may be rounded to the nearest integer, i.e., 0 and 1. This will lead to a substantially robust method for encrypting and decrypting messages.

**Table 1:** Encrypted message obtained with an encryption frequency of 62,950 Hz.

0.7956	0.7310	1.0937	0.9639	1.1013	0.9699	0.9327	0.8381
0.7758	1.2088	1.0538	1.0782	0.8784	0.9500	0.7868	1.2372

If we consider a recipient of the message using equipment with a noise leading to a deviation of 50Hz on the frequency of the decryption of the encrypted message, one obtains decrypted index components to within at most 25% of the original message (see Table 2). For illustrative purpose, we have used a large deviation of 50Hz from the encryption frequency. An easily achievable tighter control of the frequency, such as the one we have in our experiment (e.g., 1Hz) leads to a very robust method of encryption.

**Table 2:** Message obtained with a deviation of 50Hz from the encryption frequency.

− 0.2552	0.9588	0.0144	0.7950	− 0.0303	0.8222	− 0.1519	0.8961
0.0252	1.0025	0.1947	− 0.1024	0.1464	− 0.0740	0.0820	0.9424

Small variations in ${\varphi }_{12}^{(i)}$ constitute another possible source of noise as shown in the Fig. 1. For example, a significant variation of 5% in the ${\varphi }_{12}^{(i)}$ of the five phi-bits leads to decrypted index component deviating from the original message by at most 10%. Since the five phi-bits exhibit the same overall scaled continuous behavior, a simple averaging scheme could be used as a correction to significantly reduce that source of noise.

We have successfully illustrated this encoding and encryption technique using a 5 phi-bits system, chosen due to the length of our message. As phi-bits occupy the same physical domain and are strongly correlated, the scalability remains unaffected by distance, the system can accommodate *N* phi-bits with the same processing time for encryption and decryption. This scalability arises from the fact that it takes the same time to tune the driving condition of the physical system. The length of messages that can be encrypted scales exponentially as 2N, this leads to a substantial advantage for large N.

## Comparison with classical encryption

The requirement for perfectly secured cryptography is that the encrypted data and the original message should be statistically independent^17,18^. The correlation analysis was conducted between the original message and encrypted vector by sweeping the frequency ${f}_{1}$ from 62 to 64kHz. This analysis shows the non-significance of the vector (*p* > 0.05). A p-value below the conventional threshold of 0.05 indicates a lack of statistical significance between the encoded vector (original message) and the encrypted matrix. This observation highlights the efficacy of our encryption system, confirming the ability to preserve the confidentiality of the original message without introducing patterns discernable using cryptanalysis like linear and differential cryptanalysis^19^. Additionally, this means that the encrypted matrix passed the statistical test of randomness, it is unpredictable and can be reproduced reliably (i.e. running the experiment with the same conditions leads to only small variations in the encrypted data)^19^.

In the concept of classical cryptography, unicity distance is defined by^17^ as the least number of encrypted data needed for an intruder to estimate the encryption key successfully. The perfect secrecy condition states that the entropy (uncertainty in information) of the key must be greater than that of the message^17^. Here, the length of the message and the change in the driving condition of the system (key) are completely independent. It will only take a sweep of the frequency out of many possible options for *N* length of data. Consequently, the key is independent of the original message and the cipher text. Also, the indexing of the state vector is not unique and the chances of an eavesdropper with a classical computing power to predict the exact 2^*N*^ vector used for the encryption is 1 out of 2^*N*^. This will pose a great challenge for eavesdroppers with classical power even if they have much information about the cipher text. Additional security analysis of this scheme against a quantum attack will be studied in our future works.

## Conclusion

This research emphasizes the crucial requirement for strong data security in governmental and non-governmental sectors, especially with the rise of quantum computing risks. Classical cryptography has been a fundamental method for protecting confidential data by using encryption methods based on mathematical operations and secret keys. The rapid progress of quantum computing technology presents substantial challenges to classical encryption systems, as quantum computers have the capability to break modern encryption schemes that are now deemed secure.

We present the idea of logical phi-bits, utilizing the inherent nonlinearity acoustic waves in externally driven waveguides to create a scalable and versatile encryption system. Logical phi-bits, nonlinear acoustic waves, operate on principles similar to qubits in quantum computing and have the potential to transform encryption techniques by providing strongly correlated superpositions of states in the same physical domain.

We have demonstrated the viability of using multiple logical phi-bits for data encryption using a 5 phi-bit system. We achieve efficient encryption and decryption operations by manipulating the state vectors representing phi-bits superpositions through controlled changes in driving frequencies. Additionally, this research shows that the encryption processing time is constant and independent of the number of phi-bits considered for encryption. In this study, we focused on demonstrating the feasibility of an encryption scheme using a phi-bit state vector, while a comprehensive security analysis, including the transmission of the message, is recognized as crucial in the field of cryptography but remains beyond the scope of this paper and will be addressed in future work. Furthermore, our methodology allows for additional research in enhancing encryption methods through permutations of the state vector product to encode the message within the initial portion of the state vector, which could lead to the elimination of sending message indices between sender and recipient. With permutations, the challenge for a classical computer scale like 2^N^! rather than simply 2^N^ a truly daunting challenge. As quantum computing technologies advance, it is crucial for cryptographic methods to adjust correspondingly. By utilizing logical phi-bits, one can strengthen data security at speeds and scales comparable to quantum information modalities without suffering from the fragility inherent in quantum systems.

## Data Availability

The datasets generated during and/or analysed during the current study are available from the corresponding author on reasonable request.
